# Limited sensitivity and specificity of the ACR/EULAR-2019 classification criteria for SLE in JSLE?—observations from the UK JSLE Cohort Study

**DOI:** 10.1093/rheumatology/keab210

**Published:** 2021-03-04

**Authors:** Eve M D Smith, Sajida Rasul, Coziana Ciurtin, Eslam Al-Abadi, Kate Armon, Kathryn Bailey, Mary Brennan, Janet Gardner-Medwin, Kirsty Haslam, Daniel P Hawley, Steven Lane, Alice Leahy, Valentina Leone, Gulshan Malik, Devesh Mewar, Robert Moots, Clarissa Pilkington, Athimalaipet V Ramanan, Satyapal Rangaraj, Annie Ratcliffe, Phil Riley, Ethan Sen, Arani Sridhar, Nick Wilkinson, Michael W Beresford, Liza J McCann, Christian M Hedrich

**Affiliations:** 1 Department of Women’s & Children’s Health, Institute of Life Course and Medical Sciences, University of Liverpool; 2 Department of Paediatric Rheumatology, Alder Hey Children's NHS Foundation Trust Hospital, Liverpool; 3 Department of Paediatric Rheumatology, Royal Manchester Children’s Hospital, Manchester; 4 Department of Rheumatology, Centre for Adolescent Rheumatology, University College London, London; 5 Department of Rheumatology, Birmingham Children’s Hospital, Birmingham; 6 Department of Paediatric Rheumatology, Cambridge University Hospitals, Cambridge; 7 Department of Paediatric Rheumatology, Oxford University Hospitals NHS Foundation Trust, Oxford; 8 Department of Paediatric Rheumatology, Royal Hospital for Sick Children, Edinburgh; 9 Department of Child Health, University of Glasgow, Glasgow; 10 Department of Paediatrics, Bradford Royal Infirmary, Bradford; 11 Department of Paediatric Rheumatology, Sheffield Children’s Hospital, Sheffield; 12 Department of Paediatric Rheumatology, Southampton General Hospital, Southampton; 13 Department of Paediatric Rheumatology, Leeds Children Hospital, Leeds; 14 Paediatric Rheumatology, Royal Aberdeen Children’s Hospital, Aberdeen; 15 Department of Rheumatology, Liverpool University Hospitals NHS Foundation Trust, Liverpool; 16 Faculty of Health, Social Care and Medicine, Edge Hill University, Ormskirk; 17 Department of Paediatric Rheumatology, Great Ormond Street Hospital, London; 18 University Hospitals Bristol NHS Foundation Trust & Bristol Medical School, University of Bristol, Bristol; 19 Department of Paediatric Rheumatology, Nottingham University Hospitals, Nottingham; 20 Department of Paediatrics, Musgrove Park Hospital, Taunton; 21 Paediatric Rheumatology, Great North Children’s Hospital, Royal Victoria Infirmary, Institute of Cellular Medicine, Newcastle University, Newcastle upon Tyne; 22 Leicester Children’s Hospital, University Hospitals of Leicester NHS trust, Leicester; 23 Department of Paediatric Rheumatology, Guy’s & St Thomas’s NHS Foundation Trust, Evelina Children’s Hospital, London, UK

**Keywords:** SLE, paediatric, childhood, juvenile-onset SLE, classification, performance, ACR, EULAR

## Abstract

**Objectives:**

This study aimed to test the performance of the new ACR and EULAR criteria, that include ANA positivity as entry criterion, in JSLE.

**Methods:**

Performance of the ACR/EULAR-2019 criteria were compared with Systemic Lupus International Collaborating Clinics (SLICC-2012), using data from children and young people (CYP) in the UK JSLE Cohort Study (*n* = 482), with the ACR-1997 criteria used as reference standard. An unselected cohort of CYP positive for ANA (*n* = 129) was used to calculate positive/negative predictive values of the criteria.

**Results:**

At both first and last visits, the number of patients fulfilling the different classification criteria varied significantly (*P* < 0.001). The sensitivity of the SLICC-2012 criteria was higher when compared with that of the ACR/EULAR-2019 criteria at first and last visits (98% *vs* 94% for first visit, and 98% *vs* 96% for last visit; *P* < 0.001), when all available CYP were considered. The ACR/EULAR-2019 criteria were more specific when compared with the SLICC-2012 criteria (77% *vs* 67% for first visit, and 81% *vs* 71% for last visit; *P* < 0.001). Significant differences between the classification criteria were mainly caused by the variation in ANA positivity across ages. In the unselected cohort of ANA-positive CYP, the ACR/EULAR-2019 criteria produced the highest false-positive classification (6/129, 5%).

**Conclusion:**

In CYP, the ACR/EULAR-2019 criteria are not superior to those of the SLICC-2012 or ACR-1997 criteria. If classification criteria are designed to include CYP and adult populations, paediatric rheumatologists should be included in the consensus and evaluation process, as seemingly minor changes can significantly affect outcomes.

**Rheumatology key messages**

- In children, the ACR/EULAR-2019 criteria are not superior to the SLICC-2012 or the ACR-1997 criteria for SLE.
- In ANA-positive children, the ACR/EULAR-2019 criteria can result in false-positive classification.
- Paediatricians should be involved in the development of classification criteria to be applied in children.

## Introduction

JSLE is a severe, multisystem autoimmune/inflammatory disease characterized by systemic inflammation, tissue and organ damage, and the presence of autoantibodies directed against nuclear auto-antigens [1]. Presentation and outcomes vary significantly between individuals, which can complicate diagnosis, generation of evidence through clinical trials, and treatment of patients [2].

Classification criteria are an important tool for ensuring consistent case definition, particularly in relation to clinical trials. Widely accepted and used criteria for SLE were developed by the ACR in 1982 [3], and updated in 1997 (ACR-1997). Those criteria include 11 clinical and laboratory items, ≥4 being required for classification as SLE. Each element is weighted equally and (technically) patients can be classified as SLE in the absence of immunological anomalies [4]. Due to concerns that the ACR criteria may miss some SLE patients, in particular those with LN and autoantibody positivity but limited systemic involvement, the Systemic Lupus International Collaborating Clinics (SLICC-2012) group established further criteria, including 11 clinical and 6 immunological items [5]. Each criterion is weighted equally, and a score of ≥4 is required for classification as SLE. Of note, SLICC-2012 also stipulated that patients with LN and ANA and/or anti-dsDNA antibody positivity can be defined as SLE, in the absence of other clinical criteria [5]. Three studies have examined the performance of the SLICC-2012 criteria in international JSLE cohorts. All three studies demonstrated higher sensitivity for the SLICC-2012 criteria (between 92.9 and 98.7%) as compared with the ACR-1997 criteria (between 76.6 and 85.6%) [6–8]. Only one of these studies included a control group, and therefore was able to assess the specificity: they demonstrated lower specificity for SLICC-2012 (85.3%) when compared with the ACR-1997 criteria (93.4%) [8].

Recently, the ACR and the EULAR proposed a new set of classification criteria for SLE (ACR/EULAR-2019 criteria), validated in large adult SLE cohorts [9]. ANA positivity is a mandatory entry criterion (an ANA titre of ≥1:80 on human epithelial type 2 cells or equivalent positive test result). Thereafter, a weighted scoring system requires the patient to score ≥10 points to be classified as SLE. Similar to the SLICC-2012 criteria, the ACR/EULAR-2019 criteria are separated into clinical and immunological features. Based on data from adult cohorts, the ACR/EULAR-2019 criteria show better sensitivity than the ACR-1997 criteria (96% *vs* 83%) and comparable sensitivity with the SLICC-2012 criteria (97%). Specificity is the same for the ACR/EULAR-2019 and ACR-1997 criteria (both 93%) and lower for the SLICC-2012 criteria (84%) [9].

Data on performance of ACR/EULAR-2019 criteria in JSLE is limited to two relatively small cohorts that both suggested limited specificity when compared with ACR-1997 or SLICC-2012 criteria [10, 11]. These studies did not include longitudinal assessment of the ACR/EULAR-2019 criteria.

This study aimed to: (i) test performance of the ACR/EULAR-2019 classification criteria in the UK JSLE Cohort Study population longitudinally (first *vs* last visits) and in relation to age at diagnosis (pre-/peri-/post-pubertal); (ii) investigate ACR/EULAR-2019 criteria in an unrelated cohort of ANA-positive individuals; and (iii) compare the performance of the ACR/EULAR-2019 classification criteria with that of the ACR-1997 and SLICC-2012 criteria.

## Methods

### Participants

The UK JSLE Cohort Study [12], collects longitudinal clinical data from almost all UK paediatric rheumatology centres (*n* = 22) treating children and young people (CYP; up to 18 years) with JSLE. It primarily recruits patients who meet ≥4 ACR classification criteria for SLE [13]. It also recruits patients with probable lupus (fulfilling <4 ACR criteria), i.e. an experienced consultant clinician anticipates that the patient will evolve into JSLE. As such, not all patients fulfil the ACR-1997 classification criteria for SLE.

The UK JSLE Cohort Study [13] patients included in this study fulfilled the following inclusion criteria: (1) had data collected between July 2016 and January 2019 (that included SLICC-2012 classification criteria collected between these time points), (2) an ACR-1997 score of ≥2 at inclusion, and (3) aged <18 years at the time of recruitment. The performance of SLE classification criteria was tested both in CYP fulfilling ≥4 ACR-1997 classification criteria for SLE, and in CYP with a strong clinical history to suggest a diagnosis of JSLE but where <4 ACR-1997 criteria were fulfilled at recruitment to the UK JSLE Cohort Study (representing probable JSLE cases). Throughout the manuscript, where all UK JSLE Cohort patients are included in the analysis (those fulfilling ≥4 ACR-1997 classification criteria and probable cases with <4 ACR-1997 criteria at recruitment), they are described as the ‘full UK JSLE Study Cohort’.

Self-reported ethnicity information was collected according to UK National Census categorizations [14]. Data of mixed-race patients were grouped with those of the associated ethnic minority group; a category of ‘other’ was available for those not wishing to report ethnicity. The study has full ethical approval (National Research Ethics Service North West, Liverpool, UK, reference 06/Q1502/77). Research was carried out in accordance with the Declaration of Helsinki, and all patients or their legal guardians gave written confirmed consent.

### Data collected

Demographic and clinical data were collected at the patients’ first clinical assessment at the time of recruitment to the UK JSLE Cohort Study and at their last study visit, which is the final or most recent clinical assessment. The UK JSLE Cohort Study collects the paediatric adaptation of the 2004 BILAG index (pBILAG-2004) DAS at each clinical encounter [12]. It also collects the ACR-1997 classification criteria for SLE at baseline and annually. Disaggregated pBILAG scores and ACR-1997 classification criteria data were used to calculate the ACR/EULAR-2019 scores (first and last study visits). ANA positivity was defined as a titre of ≥1:80. Renal biopsy data were also obtained where available.

### ANA-positive patients presenting over a 12-month period (i.e. unselected ANA-positive control Cohort)

Clinical and laboratory data were collected from electronic patient charts of 129 CYP who, as part of an investigative work-up, were found to be ANA positive (titre of ≥1:80, between 01/2018–01/2019) and therefore fulfilled the ACR/EULAR-2019 entry criterion for SLE. Data were used to calculate ACR-1997, SLICC-2012 and ACR/EULAR-2019 scores. The electronic records of these patients were re-checked 18 months after the initial positive ANA measurement, to check whether the patients’ diagnosis had changed over time.

### Statistical analysis

Data from the UK JSLE Cohort Study were used to assess the performance of the ACR/EULAR-2019 classification criteria for SLE, primarily against the ACR-1997 criteria (the reference criteria), but also against the SLICC-2012 criteria. Data are primarily expressed descriptively (median, range, percentages and interquartile ranges). The differences between age groups, classification criteria and demographic details were compared using *χ*^2^ tests. Where comparisons were made between three different groups (e.g. age groups) and a significant difference was detected, further *χ*^2^ tests were used to determine exactly where the significant difference lay, with a Bonferroni correction being applied for multiple testing.

Sensitivity, specificity, and positive and negative predictive values (PPVs, NPVs) were calculated to assess the performance of the SLICC-2012 and ACR/EULAR-2019 classification criteria against the ACR-1997 criteria (reference criteria). In these analyses, the UK JSLE Cohort group of patients (*n* = 482) were combined with the unselected ANA-positive patients (*n* = 129), with the latter acting as a control group (total *n* = 611). Chi-squared tests were used to calculate *P*-values for the sensitivities and specificities, and the binomial exact test was used to calculate *P*-values for the PPVs and NPVs. McNemar’s test was used to assess for a difference between the ACR-1997 and ACR/EULAR2019 criteria, the ACR-1997 and SLICC-2012 criteria, and the SLICC-2012 and ACR/EULAR-2019 criteria, in the number of patients classified as having JSLE at first and last visits.

In the absence of a definitive gold standard, the level of agreement between the different criteria was also assessed using receiver operator curves (ROCs). In these analyses, the area under the curve (AUC) was calculated for the following comparisons: ACR-1997 *vs* ACR/EULAR-2019, ACR-1997 *vs* SLICC-2012, and SLICC-2012 *vs* ACR/EULAR-2019 criteria. AUC values of 1.0–0.9, 0.9–0.8, 0.8–0.7, 0.7–0.6 and 0.6–0.5 were considered to be excellent, good, fair, poor and fail, respectively [13]. Kappa coefficients were calculated to assess inter-rater agreement between the criteria. A kappa coefficient value of >0.4 was considered acceptable [15, 16]. Absolute values and CIs for the AUC and kappa coefficient are reported. All statistics were calculated using STATA 14 (StataCorp LLC, USA) and Excel (Microsoft, USA). Results were considered significant if the *P*-value was <0.05.

## Results

### UK JSLE Cohort Study participants’ clinical and demographic features

From inception to date, the UK JSLE Cohort Study has recruited 760 patients. A total of 482 patients met this study’s inclusion criteria. The median age at diagnosis was 12.8 years [interquartile range (IQR) 10.4–17.9], with a male-to-female ratio of 1:5. Data on age of onset were missing for 5 patients; therefore, of the 477 JSLE patients where age of onset was available, 50 (10%) were classified as having JSLE with disease onset at <8 years of age (pre-pubertal), 268 (56%) at 8–13 years (peri-pubertal), and 159 (33%) at 14–18 years of age (adolescent). Median follow-up was 39 months (IQR 18–195). Ethnicity data were missing for 10 patients. Of the 472 where ethnicity was known, 242 (50%) of patients were White Caucasian, 140 (29%) were South Asian, 73 (15%) were Black African/Caribbean and 17 (4%) were of a mixed ethnic background. ANA positivity at first visit was highest in patients presenting between 14–18 years (95%) compared with other age groups (<8 years: 88%; 8–13 years: 93%). The demographic and clinical information is summarized in Table 1.

**Table 1:** Demographic details of the full UK JSLE Cohort Study, and those scoring ≥4 ACR-1997 criteria

Demographics	Full UK JSLE Cohort Study^a^ *n* = 482 (%)	ACR-1997 ≥ 4	
		First visit, *n* = 385 (%)^b^	Last visit, *n* = 427 (%)^c^
Ethnicity			
White Caucasian	242 (51%)	180 (47%)	209 (49%)
Black African/Caribbean	73 (15%)	58 (15%)	66 (15%)
South Asian	140 (29%)	119 (31%)	128 (30%)
Other	17 (4%)	14 (4%)	15 (4%)
Gender^a^			
Female	402 (83%)	318 (83%)	357 (93%)
Male	74 (15%)	61 (16%)	64 (17%)
Age			
Median age at diagnosis (IQR)	12.8 (10.4–17.9)	12.9 (10.7–17.9)	12.8 (10.5–18.0)
Numbers of patients in different age groups			
<8 years	50 (10%)	37 (10%)	43 (10%)
8–13 years	268 (56%)	218 (57%)	242 (57%)
14–18 years	159 (33%)	127 (33%)	138 (32%)
ANA positivity according to age group			
<8 years	44 (88%)	34 (92%)	42 (98%)
8–14 years	249 (93%)	205 (94%)	233 (96%)
14–18 years	151 (95%)	125 (98%)	135 (98%)

Full UK JSLE Study Cohort includes patients fulfilling ≥4 ACR-1997 criteria and those fulfilling 2–3 ACR-1997 criteria. Numbers of patients and percentages shown. ^a^Data missing for the full JSLE Study Cohort patients is as follows: (a) 5 patients for age, (b) 10 patients for ethnicity, (c) 38 patients for ANA, (d) 6 patients for gender. ^b^Data missing for the ACR-1997 ≥ 4 cohort patients at first visit is as follows: (a) 3 patients for age, (b) 14 patients for ethnicity, (c) 21 patients for ANA and (d) 6 patients for gender in the subgroup of patients ACR-1997 ≥ 4. ^c^Data missing for the ACR-1997 ≥ 4 cohort patients at last visit is as follows: (a) 4 patients for age, (b) 9 patients for ethnicity, (c) 17 patients for ANA and (d) 5 patients for gender in the subgroup of patients ACR-1997 ≥ 4. *P*-values for comparisons made between each demographic category within the full cohort (*n* = 482) are <0.001 for ethnicity, <0.001 for gender, <0.001 for age groups at diagnosis, and 0.24 for ANA positivity according to age. *P*-values for differences between the first and last groups were not calculated, because the patients form the same overall group and are therefore not independent. Statistical analyses comparing the demographic details between the full JSLE Study Cohort *vs* those fulfilling ≥4 ACR-1997 criteria at the first and last visit are not undertaken, because there is overlap in the patients included in the different subgroups. ACR-1997: ACR 1997 revised version of criteria. IQR: interquartile range.

### Participants fulfilling SLE classification criteria at first and last visits

**Fig. 1:**
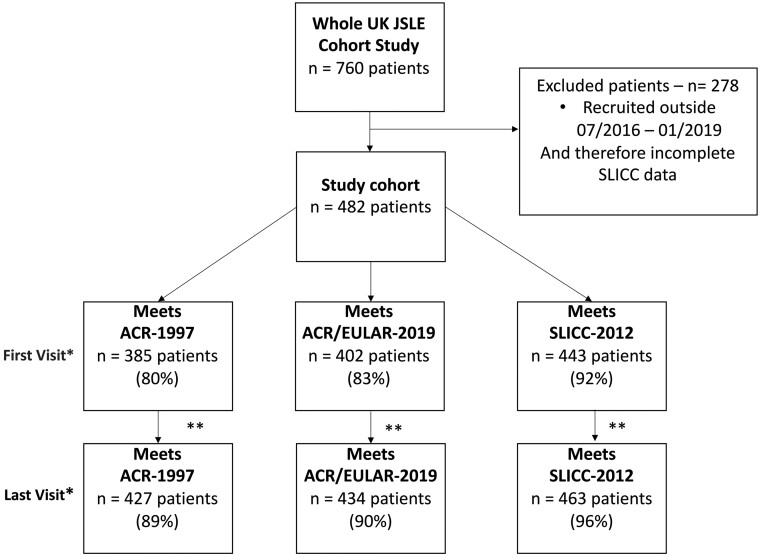
Patients classified as having JSLE at first and last visit using three sets of criteria Full UK JSLE Cohort Study: 482 patients. Section sign indicates Chi squared test used to calculate *P*-values for differences in the numbers classified using each set of criteria. *P*-value at first (*P* = 0.007) and last visit (*P* < 0.001). Hash symbol indicates McNemar’s test was used to calculate the *P*-values for differences in the number classified initially and finally by individual classification criteria: ACR-1997, *P* < 0.001, ACR/EULAR-2019, *P =* 0.0003; SLICC, *P* = 0.0001.

The number of UK JSLE Cohort Study participants fulfilling the ACR-1997, SLICC-2012 and ACR/EULAR-2019 classification criteria at the time of their first and last visits are displayed in Fig. 1. At first visit, 385/482 (80%) patients fulfilled the ACR-1997 criteria for SLE, 402/482 (83%) fulfilled the ACR/EULAR-2019 criteria and 443/482 (92%) fulfilled the SLICC-2012 criteria. By the last visit, 427/482 (89%) patients fulfilled the ACR-1997 criteria, 434/482 (90%) fulfilled the ACR/EULAR-2019 criteria and 463/482 (96%) fulfilled the SLICC-2012 criteria. There was a significant increase in the number of patients classified as SLE between the first and last visits by all classification criteria (all *P*< 0.001). At both first and last visits, the number of patients fulfilling the different classification criteria (ACR-1997, SLICC-2012 and ACR/EULAR-2019) varied significantly (*P* < 0.001 at both first and last visits).

At the first visit, 30 (6%) patients who would otherwise have scored ≥10 using the ACR/EULAR-2019 criteria for SLE were ANA negative and therefore did not fulfil the ACR/EULAR-2019 entry criterion for SLE [despite 18/30 (60%) fulfilling either ACR-1997 or SLICC-2012 criteria]. Of these patients, 15/30 (50%) subsequently developed ANA positivity, and therefore met the ACR/EULAR-2019 criteria by their last visit. Further information on the individual criteria fulfilled by patients who met the classification criteria threshold for one set of criteria, but not the others, at their first visit is shown in Supplementary Table S1, available at *Rheumatology* online.

### Clinical and laboratory criteria fulfilled

Individual clinical and immunological items fulfilled from the three different classification criteria at first and last presentations are displayed in Supplementary Table S2, available at *Rheumatology* online. Of note, across the UK JSLE Cohort Study, the proportion of ANA-positive individuals increased from the first visit (448/482, 93%) to the last visit (463/482, 96%; *P* < 0.001), increasing the number of individuals fulfilling the ACR/EULAR-2019 entry criterion. At the first visit, 10 patients exhibited grade III or IV nephritis on biopsy, while testing negative for ANA at inclusion/first visit. Three patients continued to be ANA negative at the time of their last visit. A negative ANA would exclude these 10 patients at their first visit and 3 patients at their last visit from classification of SLE using the ACR/EULAR-2019 criteria.

### SLE classification criteria in relation to age at diagnosis within the full UK JSLE Study Cohort (*n* = 482)

Age-specific differences in clinical presentation and laboratory findings have previously been demonstrated in CYP with JSLE [17]. To address the question of whether the number of classification variables differed according to age, JSLE patients were subdivided into the following groups: pre-pubertal (<8 years), peri-pubertal (8–13 years) and adolescent/post-pubertal (14–18 years) (Table 2). For the ACR/EULAR-2019 criteria, a significant difference was seen in the proportion of patients fulfilling the criteria according to age, at both first (*P* = 0.02) and last visits (*P* = 0.001). At first visit, a lower proportion of pre-pubertal patients (32/50, 64%) fulfilled the ACR/EULAR-2019 criteria as compared with both peri-pubertal (8–13 years; 219/268, 81%, *P* = 0.04) and adolescent patients (14–18 years; 126/159, 79%; *P* = 0.002, Table 2). This likely relates to the observation that ANA positivity was numerically highest at diagnosis in adolescent patients (14–18 years: 151/159, 95%) when compared with other age groups (<8 years: 44/50, 88%; 8–13 years: 249/268, 93%) (Table 1). This difference was not seen using either the ACR-1997 or SLICC-2012 criteria (*P*-values > 0.05).

**Table 2:** Number of UK JSLE Cohort patients fulfilling the different classification criteria for SLE (by age)

	Number of patients fulfilling the ACR-1997 criteria^a^		Number of patients fulfilling the ACR/EULAR-2019 criteria^a^		Number of patients fulfilling the SLICC-2012 criteria^a^	
	First visit *n* (%)	Last visit *n* (%)	First visit *n* (%)	Last visit *n* (%)	First visit *n* (%)	Last visit *n* (%)
Pre-pubertal <8 years (*n* = 50)	37 (74%)	43 (86%)	32 (64%)	35 (70%)	44 (88%)	48 (96%)
Peri-pubertal 8–13 years (*n* = 268)	218 (81%)	242 (90%)	219 (81%)	239 (89%)	249 (93%)	256 (96%)
Adolescent 14–18 years (*n* = 159)	127 (80%)	138 (87%)	126 (79%)	133 (84%)	144 (91%)	155 (97%)
*P*-value	0.5	0.4	0.02*	0.001**	0.2	0.7

Numbers displayed are those patients fulfilling the different classification criteria, with percentages in brackets. Overall, patient age was not available for 5/482 UK JSLE Cohort Study patients. ^a^Of the patients fulfilling ≥4 ACR-1997 revised criteria for classification of SLE (*n* = 385 at first visit and *n* = 427 at last visit), age was unknown in 3 patients at the first visit and 4 patients at the last visit. Of patients scoring ≥10 using the ACR/EULAR-2019 criteria (*n* = 402 at first visit and *n* = 434 at last visit), age was unknown in 4 patients at the first visit and 4 patients at the last visit. Of the patients fulfilling ≥4 SLICC-2012 criteria (*n* = 441 at the first visit and *n* = 463 at the last visit), age was unknown in 4 patients at the first visit and 4 patients at the last visit. ACR-1997: ACR 1997 revised version. *χ*^2^d tests used to calculate *P*-values when comparing the proportion of patients fulfilling different criteria, for individual age groups at an individual visit (either first or last visit). From *post* *hoc* analysis, one asterisk indicates a significant difference between the pre-pubertal and peri-pubertal age groups (*P* = 0.04), and the pre-pubertal and adolescent age groups (*P* = 0.002), and two asterisks indicate a significant difference between the pre-pubertal and peri-pubertal age groups (*P* = 0.05) and the pre-pubertal and adolescent age groups (*P* = 0.05).

### Performance of the SLE classification criteria in an unselected paediatric population testing positive for ANA and the full UK JSLE Study Cohort combined

To test the sensitivity, specificity, PPVs and NPVs of the classification criteria in ANA-positive CYP independent of their final diagnosis, the ACR-1997 criteria were used as the reference criteria in an unselected population of CYP who tested positive for ANA at Alder Hey Children’s Hospital over a 12 month period (*n* = 129), combined with 482 CYP from the UK JSLE Cohort Study (*n* = 611 total). The unselected ANA-positive CYP cohort consisted of 92 females and 37 males, with a median age of 11 years (IQR 7–17) (Supplementary Table S3, available at *Rheumatology* online).

At both the first and last visits, sensitivity of the SLICC-2012 criteria (both 98%) was comparable with that of the ACR-1997 criteria, and higher when compared with the ACR/EULAR-2019 criteria (first visit: 94%, last visit: 96%, both *P* < 0.001). Conversely, specificity of the SLICC-2012 criteria was significantly lower compared with that of the ACR/EULAR-2019 criteria at both first (SLICC-2012: 67% *vs* ACR/EULAR-2019: 77%, *P* < 0.001) and last visits (SLICC-2012: 71% *vs* ACR/EULAR-2019: 81%, *P* < 0.001).

The proportion of CYP fulfilling the classification criteria who were correctly identified as JSLE (PPV, based on the ACR-1997 reference criteria) was higher using the ACR/EULAR-2019 criteria compared with when using the SLICC-2012 criteria at both first and last visits (88% at first and 93% at last visit for the ACR/EULAR-2019 criteria, *vs* 84% at first and 89% at last visit for the SLICC-2012 criteria, Table 3). Conversely, the proportion of CYP not fulfilling the criteria and correctly identified as not having JSLE (NPV) was lower for the ACR/EULAR-2019 criteria compared with the SLICC-2012 criteria (87% at first and 89% at last visit for ACR/EULAR-2019, *vs* 95% at both first and last visit for SLICC-2012).

**Table 3:** Sensitivity, specificity, and positive and negative predictive values of ACR/EULAR-2019 and SLICC-2012 criteria

Classification criteria	Sensitivity		Specificity		Positive predictive value		Negative predictive value	
	First visit (%)	Last visit (%)	First visit (%)	Last visit (%)	First visit (%)	Last visit (%)	First visit (%)	Last visit (%)
ACR/EULAR-2019	94	96	77	81	88	93	87	89
SLICC-2012	98	98	67	71	84	89	94	94
*P*-values	<0.001 *	<0.001 *	<0.001 *	<0.001 *	<0.001 **	<0.001 **	<0.001 **	<0.001 **

The ACR-1997 criteria were used as reference criteria for calculation of sensitivities, specificities, and positive and negative predictive values. *P-values were calculated using the *χ*^2^d test, comparing the sensitivities or specificities obtained by the different classification criteria (ACR/EULAR-2019 *vs* SLICC-2012) at each time point. **P-values calculated using a binomial exact test, and relating to positive predictive value and negative predictive value for each set of criteria.

### Level of agreement between classification criteria

In the absence of a gold standard test for JSLE, ROC curves and kappa coefficient analysis were used to assess levels of agreement between the ACR/EULAR-2019 criteria and the previous criteria (Table 4). When the ACR-1997 criteria were used as the reference criteria to classify patients as having JSLE, the AUC for the ACR/EULAR-2019 criteria was 0.78 (CI: 0.73, 0.83). The kappa coefficient for inter-rater agreement between the ACR-1997 and the ACR/EULAR-2019 criteria was 0.58 (CI: 0.53, 0.63). When the SLICC-2012 criteria were used as the reference criteria to classify CYP as having JSLE, the AUC for the ACR/EULAR-2019 criteria was 0.89 (CI: 0.75, 0.90), and the kappa coefficient for inter-rater agreement between the two criteria was 0.76 (CI: 0.69, 0.78). This demonstrated variable agreement between the different criteria, with the strongest agreement being between the ACR/EULAR-2019 and SLICC-2012 criteria.

**Table 4:** Level of agreement between classification criteria

Reference criteria	Comparator criteria	AUC (CI)	Kappa coefficient (CI)
ACR-1997	ACR/EULAR-2019	0.78 (0.73, 0.83)	0.58 (0.53, 0.63)
	SLICC-2012	0.63 (0.54, 0.67)	0.37 (0.27, 0.41)
SLICC-2012	ACR/EULAR-2019	0.89 (0.75, 0.90)	0.76 (0.69, 0.78)

ACR-1997: ACR 1997 revised version, AUC from ROC curves generated using data from the full UK JSLE Cohort Study, *n* = 482. AUC: area under the curve.

### False-positive classification of CYP using the ACR/EULAR-2019 in an unselected CYP cohort testing positive for ANA

A total of 6/129 (5%) individuals in the aforementioned cohort tested positive for ANA, despite having an alternative diagnosis (false positives). Of these, 5/6 met the ACR/EULAR-2019 criteria, 4/6 met the SLICC-2012 criteria, and 2/6 met the ACR-1997 criteria for SLE (Table 5). Two patients fulfilled all three SLE classification criteria, including one patient with RNP-positive mixed connective tissue disease and one patient with biopsy-proven renal dysplasia. Two patients exclusively fulfilled the ACR/EULAR-2019 criteria, including one patient diagnosed with Cornelia de Lange syndrome and one with IgA vasculitis. One patient diagnosed with JDH met both the ACR/EULAR-2019 and the SLICC-2012 criteria, and one patient with LPS-responsive beige-like anchor protein (*LRBA*) gene mutation with idiopathic thrombocytopenic purpura and hypogammaglobulinaemia met the SLICC-2012 classification criteria.

**Table 5:** False-positive classification of SLE in unselected ANA-positive control cohort

	Individual patients and diagnosis at the time of analysis	Clinical features	Classification criteria scores		
			ACR/EULAR-2019 score (*n* = 5)	SLICC-2012 score (*n* = 4)	ACR-1997 score (*n* = 2)
1	MCTD (RNP +ve)	Anti-dsDNA positivity, low complement, lymphopenia and pericardial effusion	21	6	4
2	Renal dysplasia^a^	Mouth ulcers, urine albumin creatinine ratio of >300 mg/mmol, leukopoenia and low complement	13	4	4
3	Cornelia de Lange syndrome with OA	ANA positivity, OA and low complement	10	N/A	N/A
4	IgA vasculitis	ANA positivity, arthritis and proteinuria	10	N/A	N/A
5	JDM	ANA positivity, malar rash, arthritis and low C4	10	4	N/A
6	*LRBA* gene mutation with ITP and hypo-gammaglobulinaemia	ANA positivity, alopecia, thrombocytopenia, DAT positivity	N/A	5	N/A

ACR-1997: ACR 1997 revised version, classified as SLE if score ≥4 points. ACR/EULAR-2019, classified as SLE if score ≥10 points. SLICC-2012, classified as SLE if score ≥4 points. ^a^Renal dysplasia was demonstrated on biopsy, with no inflammation demonstrated and negative immunofluorescence. The electronic records of these patients were rechecked 18–24 months after the initial positive ANA measurement, confirming that none of the initial diagnoses had changed over this time period. DAT: direct antiglobulin test; MCTD: mixed connective tissue disease; RNP +ve : RNP antibody positive; LRBA: LPS-responsive beige-like anchor protein; ITP: idiopathic thrombocytopenia purpura; N/A: non-applicable (criteria threshold not met).

## Discussion

Classification criteria are important and accepted tools allowing selection of homogeneous patient cohorts for clinical trials. By definition, classification criteria therefore aim for high specificity while allowing reduced sensitivity [3, 4, 18, 19]. This discriminates classification from diagnostic criteria, which aim for high sensitivity while accepting reduced specificity to not miss patients in the diagnostic process [18]. Recently published ACR/EULAR-2019 criteria for SLE were the result of a consensus process of adult rheumatologists, aiming at a homogeneous case definition of SLE patients, not primarily considering potential differences between JSLE and adult-onset disease. Paediatric rheumatologists were not involved in the process, and JSLE cohorts were also not included in performance testing. Therefore to date, it remains largely unclear whether these new criteria perform sufficiently well in CYP with JSLE [9, 20].

Two recent studies have assessed the performance of ACR/EULAR-2019 criteria in JSLE [10, 11]. The first study included 122 JSLE patients and 89 controls (ANA positive with other rheumatic diseases). Using an ACR/EULAR-2019 criteria cut-off score of ≥10, the new criteria were less specific at the time of the first visit (67.4%) than both the ACR-1997 (83.2%) and the SLICC criteria (80.9%). For sensitivity, the new ACR/EULAR-2019 criteria scored better than ACR 1997 (87.7% *vs* 70.5%) and worse than the SLICC-2012 criteria (89.3%). The authors assessed additional cut-off points for the new ACR/EULAR-2019 score, showing a score of ≥13 resulting in increased specificity, and improved PPV and cut-off point accuracy [11].

The second study included 112 SLE patients aged 2–21 years (with JSLE and adult-onset SLE) and 105 controls aged 1–19 years (with other rheumatic diseases). The rheumatologist’s diagnosis of SLE served as the reference standard criterion. The authors showed the ACR/EULAR-2019 criteria to have higher sensitivity (85% *vs* 72%; *P* = 0.023) and similar specificity (83% *vs* 87%; *P* = 0.456) when compared with the 1997-ACR criteria. On examining the ACR/EULAR-2019 classification summary scores according to ethnicity, the absolute scores were higher in non-White than White patients (22 + 10 *vs* 17 + 9; *P* < 0.01). Sub-analysis showed sensitivity of the criteria was not influenced by patient ethnicity, age or gender [10].

In this present study including a markedly larger national study population (the UK JSLE Cohort Study), differences between the ACR-1997 and SLICC-2019 *vs* the ACR/EULAR-2019 criteria were mainly caused by the absence of the entry criterion, ANA positivity, affecting a total of 30 CYP (6%). Indeed, higher frequencies of ANA-negative patients diagnosed and/or classified as having JSLE have been reported previously, and are therefore a concern in relation to the ACR/EULAR-2019 criteria [17]. ANA negativity, especially in young JSLE patients, may be associated with a strong genetic contribution to disease pathology (e.g. monogenic causes or an increased number of risk alleles), which may cause systemic inflammation and tissue damage (initially) in the absence of autoantibodies. Indeed, a higher relative prevalence of genetic forms of SLE (recently estimated to be ∼7% [21]) and a higher number of risk alleles within individuals across the remaining JSLE patient population [22] likely contribute to more severe clinical phenotypes with increased disease activity and organ damage, and higher proportions of ANA-negative patients when compared with adult-onset SLE [23]. Of note, over time, 50% of initially ANA-negative JSLE patients in the UK JSLE Cohort Study developed ANA positivity, and therefore at their last visit also met the ACR/EULAR-2019 criteria. While one could argue that this is of benefit when selecting homogeneous populations for clinical trials, it creates problems for JSLE patients in whom their condition is evolving and who develop autoantibody positivity over time [17].

Another concern is that in the in absence of widely agreed diagnostic criteria for SLE, many healthcare professionals use classification criteria to aid diagnosis. Using the ACR/EULAR-2019 criteria to do this would result in a significant proportion of JSLE patients (especially ANA-negative patients) that may be missed. Unfortunately, this will mostly affect young JSLE patients, in whom diagnosis can already be delayed [24]. Particularly among pre-pubertal JSLE patients (pre-pubertal, <8 years), fewer individuals fulfilled the ACR/EULAR-2019 criteria when compared with the ACR-1997 and SLICC-2012 criteria [17].

Using a combined cohort including the UK JSLE Cohort Study participants and the unselected ANA-positive CYP to calculate specificity, sensitivity and predictive values, based on the ACR-1997 criteria as reference criteria, reduced sensitivity was calculated for the ACR/EULAR-2019 criteria compared with the SLICC-2012 criteria, while specificity was higher in the ACR/EULAR-2019 criteria compared with the SLICC-2012 criteria. This confirms findings from above in a larger cohort including additional differential diagnoses, and indicates that inclusion of ANA as an entry criterion may reduce sensitivity, while potentially increasing specificity. Thus, if (incorrectly) used to diagnose patients, the ACR/EULAR-2019 criteria may miss individuals and/or delay diagnosis in CYP who develop autoantibodies later in disease, including those cases resulting from monogenic disease causes [17].

As classification criteria aim at high specificity while potentially accepting slightly reduced sensitivity, we investigated an unselected cohort of ANA-positive CYP. Five patients were falsely classified as having JSLE using the ACR/EULAR-2019 criteria, while this was the case in four patients when using the SLICC-2012 criteria and in two individuals when using the ACR-1987 criteria. Thus, specificity of the EULAR/ACR-2019 criteria may indeed be limited when compared with that of other sets of criteria, resulting in false-positive results. Other immune complex–mediated conditions with ANA positivity and renal involvement are of particular concern (e.g. IgA vasculitis) [25].

Taken together, while it is challenging to propose changes to consensus-based classification criteria developed following a stringent process, including access to patient data and clinical findings across large (adult) SLE cohorts, from a paediatric perspective, main concerns in relation to false-positive or -negative classification include (i) ANA antibody positivity as an entry criterion (missing a significant proportion of young JSLE patients (17]), and (ii) the combination of ANA positivity and immune complex vasculitis triggering classification as SLE (as this may be present in IgA vasculitis, a relatively common condition in childhood).Thus, additional studies further investigating the performance of the ACR/EULAR-2019 classification criteria in multi-ethnic cohorts, across ages, and at different disease stages are warranted. Inclusion of subcohorts of CYP with different systemic inflammatory diseases will be critical for reliably evaluating specificity and sensitivity.

The absence of widely accepted diagnostic tools for JSLE meant that the ACR-1997 criteria needed to be used as a reference standard. Particular strengths of this cohort are the availability of longitudinal data in a national cohort, allowing assessment of classification criteria performance at different disease stages (first *vs* last visits). This, and the significantly larger sample size are key enhancements when compared with the two previous studies comparing the ACR/EULAR-2019 criteria with the ACR-1997 and SLICC-2012 criteria in JSLE cohorts. Future assessment of how these criteria perform in an international cohort of JSLE patients is also warranted.

## Conclusions

Based on observations in a large national JSLE cohort (the UK JSLE Cohort Study), the ACR/EULAR-2019 criteria miss a significant proportion of pre-pubertal JSLE patients, mostly because of the absence of ANA positivity. Performance improves with age, and sensitivity (initially reduced) is comparable with that of the SLICC-2012 criteria at the last visit. Overall, the specificity is higher when compared with the SLICC-2012 criteria. However, concerns remain due to more false positives being seen using the ACR/EULAR-2019 criteria. Given the rarity of JSLE, some clinicians will have limited experience in making the diagnosis of JSLE and may rely on classification criteria to aid diagnosis. Doing this with the ACR/EULAR-2019 criteria, a significant proportion of JSLE patients (especially ANA-negative patients) may be initially missed, leading to diagnostic delay, morbidity and potentially mortality. If classification criteria are designed to include paediatric and adult populations, paediatric specialists should be consulted and included in the consensus and evaluation process, as seemingly minor differences can affect outcomes.

## Supplementary Material

keab210_Supplementary_Data
