# Supplementary material for: Limited sensitivity and specificity of the ACR/EULAR-2019 classification criteria for SLE in JSLE?—observations from the UK JSLE Cohort Study
Source: Rheumatology (Oxford). 2021 Mar 4;60(11):5271–81. doi: 10.1093/rheumatology/keab210 (PMC8566265; doi:10.1093/rheumatology/keab210)
Supplement: keab210_Supplementary_Data [file keab210_supplementary_data.docx]

**Supplementary Table S1: JSLE patients meeting one set of classification criteria at first visit, but not another set**

| **Clinical/laboratory features** | **ACR-1997 criteria met (but not SLICC-2012 or ACR/EULAR-2019)^¶^** | **ACR/EULAR-2019 criteria met (but not SLICC-2012 or ACR-1997)^ƚ^** | **SLICC-2012 criteria met (but not ACR-1997 or ACR/EULAR-2019)^β^** |
| --- | --- | --- | --- |
| **Malar Rash** | 9/19 (47%) | 2/11 (18%) | 5/21 (24%) |
| **Discoid Lupus** | 1/19 (5%) | 0 | 2/21 (10%) |
| **Photosensitivity** | 10/19 (53%) | 0 | 5/21 (24%) |
| **Oral/nasal ulcer** | 11/19 (58%) | 0 | 3/21 (14%) |
| **Non-erosive arthritis** | 5/19 (26%) | 5/11 (45%) | 5/21 (24%) |
| **Serositis** | 0 | 0 | 0 |
| **Nephritis** | 0 | 3/11 (27%) | 0 |
| **Neurological disorder** | 0 | 0 | 1/21 (5%) |
| **Haematologic disorder** | 5/19 (26%) | 4/11 (36%) | 10/21 (48%) |
| **Anti-dsDNA** | 2/19 (11%) | 5/11 (45%) | 4/21 (19%) |
| **Anti-smith** | 1/19 (5%) | 3/11 (27%) | 0 |
| **Anti-Phospholipid antibodies** | 4/19 (21%) | 0 | 5/21 (24%) |
| **Low complement** | 10/19 (53%) | 2/11 (18%) | 16/21 (76%) |

¶Total number of patients fulfilling ACR-1997, but not SLICC-2012 or ACR/EULAR criteria = 19; ƚTotal number of patients fulfilling ACR/EULAR-2019 but not SLICC-2012 or ACR-1997 = 11. βTotal number of patients fulfilling SLICC-2012 but not ACR-1997 or ACR/EULAR-2019 criteria = 21. ACR-1997 = American College of Rheumatology 1997 revised version, ACR/EULAR-2019 = American College of Rheumatology/ European League Against Rheumatism 2019, SLICC-2012 = Systemic Lupus International Collaborating Clinics 2012.

**Supplementary Table S2: Clinical and immunological criteria fulfilled by full UK JSLE Cohort Study participants.**

| **Clinical characteristics** | **ACR 1997**  **(score ≥4)** | | **ACR/EULAR-2019 (score ≥10)** | | **SLICC-2012**  **(score ≥4)** | | **P-value**  **first visit^*^** | **P-value last visit^**^** |
| --- | --- | --- | --- | --- | --- | --- | --- | --- |
|  | **First visit n=385 (%)** | **Last visit n=427 (%)** | **First**  **visit n=402**  **(%)** | **Last**  **visit**  **n=434 (%)** | **First visit**  **n=441 (%)** | **Last**  **visit**  **n= 463 (%)** |  |  |
| **Malar rash** | 238 (62%) | 301 (70%) | 228  (57%) | 245 (56%) | 246 (56%) | 303 (65%) | 0.18 | **<0.001** |
| **Discoid lupus** | 38 (10%) | 65  (15%) | 37  (9%) | 58 (13%) | 39  (9%) | 65 (14%) | 0.88 | 0.48 |
| **Photosensitivity** | 97 (25%) | 160 (37%) | 86  (21%) | 125 (29%) | 103 (23%) | 160 (35%) | 0.44 | **0.02** |
| **Oral/nasal ulcer** | 134 (35%) | 188 (44%) | 109  (27%) | 129 (30%) | 138 (31%) | 190  (41%) | 0.06 | **<0.001** |
| **Non-erosive arthritis** | 244 (63%) | 287 (67%) | 239  (59%) | 262 (60%) | 266 (60%) | 292 (63%) | 0.29 | **<0.001** |
| **Serositis (pericarditis and/or pleural effusion)** | 64 (17%) | 91 (21%) | 65  (16%) | 86 (20%) | 66 (15%) | 91 (20%) | 0.33 | 0.80 |
| **Nephritis** | 138 (36%) | 166 (39%) | 137  (34%) | 170 (39%) | 136 (31%) | 167 (36%) | 0.30 | 0.28 |
| **Neurologic disorder** | 24  (6%) | 37  (9%) | 23  (6%) | 34  (8%) | 22  (5%) | 39  (8%) | 0.73 | **0.03** |
| **Haematological**  **Haemolytic anaemia**  **Leukopenia**  **Lymphopenia**  **Thrombocytopenia** | 92  (24%)  99 (26%)  180 (47%)  54 (14%) | 110 (26%)  132 (31%)  226 (53%)  83 (19%) | 93  (23%)  102  (25%)  166  (41%)  78  (19%) | 111 (26%)  120 (28%)  194 (45%)  96 (22%) | 97 (22%)  112 (25%)  196 (44%)  86 (20%) | 108 (23%)  143 (31%)  239 (52%)  94 (20%) | 0.07  0.99  0.30  **0.02** | 0.64  0.78  **0.03**  0.61 |
| **Immunological characteristics** | | | | | | | | |
| **ANA positive** | 367 (95%) | 414 (97%) | 402  (100%) | 434  (100%) | 421  (95%) | 453  (98%) | **0.02** | **0.002** |
| **Anti – dsDNA** | 266 (69%) | 313 (73%) | 264  (66%) | 306 (71%) | 294 (67%) | 330 (71%) | 0.58 | 0.64 |
| **Anti – Smith** | 80 (21%) | 107 (25%) | 78  (19%) | 108 (25%) | 95 (22%) | 117 (25%) | 0.74 | 0.97 |
| **Anti-Phospholipid antibodies** | 83 (22%) | 113 (26%) | 76  (19%) | 117 (27%) | 97 (22%) | 130 (28%) | 0.45 | 0.85 |
| **Low Complement**  **C3**  **C4**  **Both** | 163 (42%)  80 (21%)  156 (41%) | 278 (65%)  169 (40%)  267 (63%) | 163  (41%)  90  (22%)  157  (39%) | 284 (65%)  121 (28%)  272 (63%) | 175 (40%)  103 (23%)  166 (38%) | 288 (62%)  122 (26%)  274 (59%) | 0.73  0.67  0.70 | 0.54  **<0.001**  0.47 |

Numbers of patients fulfilling the different criteria at first and last visit are presented throughout the table, with the percentage of patients in brackets. Based on published criteria, an ACR-1997 score ≥4, ACR/EULAR-2019 scores ≥10, and SLICC-2012 score ≥4resulted in classification as SLE. *Comparison of the proportion of patients presenting with each clinical or immunological criterion considered by ACR-1997, ACR/EULAR-2019 and SLICC-2012 at first visit. ^**^Comparison of the proportion of patients presenting with each clinical or immunological criterion considered by ACR-1997, ACR/EULAR-2019 and SLICC-2012 at last visit. The criteria fulfilled are cumulative (at any point in time). dsDNA = double stranded DNA, ANA = anti-nuclear antibody, ACR-1997 = American College of Rheumatology 1997 revised version, ACR/EULAR-2019 = American College of Rheumatology/ European League Against Rheumatism 2019, SLICC-2012 = Systemic Lupus International Collaborating Clinics 2012. P-values calculated using chi-squared test for differences in scores at either first and last visit when comparing between all three sets of criteria.

**Supplementary table S3: Demographic of unselected ANA positive patients**

| **Demographics** | ***“unselected” CYP cohort testing positive for ANA***  **n=129 (%)** |
| --- | --- |
|  |  |
| **Gender** | |
| **Female** | 92 (71%) |
| **Male** | 37 (29%) |
| **Age at presentation** | 11 [7 – 17] |
| **<8 years** | 39 (30%) |
| **8-13 years** | 54 (42%) |
| **14-18 years** | 36 (28%) |

Unselected CYP cohort includes all patients presenting to Alder Hey Children’s Hospital who have had an ANA test undertaken as a part of their assessment and tested positive for ANA (titre of at least 1:80), between January 2018 – January 2019. There were significantly more females than males in the unselected CYP cohort (<0.001). There was also a significant difference in the age of presentation, with significantly more patients presenting between 8-13 years (p=0.02) as compared to 14-18 years. CYP = children and young people, ANA = antinuclear antibodies.
